# Design of vacuum annealing furnace temperature control system based on GA-Fuzzy-PID algorithm

**DOI:** 10.1371/journal.pone.0293823

**Published:** 2023-11-29

**Authors:** Jintao Meng, Haitao Gao, Mixue Ruan, Hai Guo, Xiaojie Zhou, Di Zhang

**Affiliations:** School of Electrical and Electronic Engineering, Anhui Science and Technology University, Bengbu, China; National Institute of Technology Silchar, India, INDIA

## Abstract

As is well known, the metal annealing process has the characteristics of heat concentration and rapid heating. Traditional vacuum annealing furnaces use PID control method, which has problems such as high temperature fluctuation, large overshoot, and long response time during the heating and heating process. Based on this situation, some domestic scholars have adopted fuzzy PID control algorithm in the temperature control of vacuum annealing furnaces. Due to the fact that fuzzy rules are formulated through a large amount of on-site temperature data and experience summary, there is a certain degree of subjectivity, which cannot ensure that each rule is optimal. In response to this drawback, the author combined the technical parameters of vacuum annealing furnace equipment, The fuzzy PID temperature control of the vacuum annealing furnace is optimized using genetic algorithm. Through simulation and comparative analysis, it is concluded that the design of the fuzzy PID vacuum annealing furnace temperature control system based on GA optimization is superior to fuzzy PID and traditional PID control in terms of temperature accuracy, rise time, and overshoot control. Finally, it was verified through offline experiments that the fuzzy PID temperature control system based on GA optimization meets the annealing temperature requirements of metal workpieces and can be applied to the temperature control system of vacuum annealing furnaces.

## 1. Introduction

As is well known, heat treatment is one of the fundamental processes in mechanical manufacturing. Heat treatment is of great significance in improving the mechanical properties of materials, eliminating residual stress, improving the internal quality of products, improving their machinability, and extending the service life of products. Vacuum annealing is a key process for heat treatment of metal materials and workpieces, and vacuum annealing furnaces are the main heating equipment for vacuum annealing [1–3]. In vacuum annealing, there are often characteristics such as time-varying, nonlinear, and time-delay, and strong coupling occurs between the temperatures of various temperature zones and between the annealing temperature and the vacuum degree in the furnace during the heating process, which increases the difficulty of temperature control [4]. The precision of temperature control during the vacuum annealing process directly affects the performance and quality of the product, which requires a precise and mature temperature control strategy for the vacuum annealing furnace temperature control system [5–7]. Traditional temperature control systems generally use manual methods based on on-site experience and control theory for control [8, 9]. Many annealing furnaces still rely on manual and conventional instruments, combined with traditional PID control strategies for temperature control. Due to the characteristics of annealing furnace temperature control such as multiple temperature zones, large volume inside the furnace, and large disturbance in the control process [10, 11]. if the temperature control strategy is not perfect, it will increase the labor intensity of workers, and the production efficiency is low, making it difficult to ensure production safety.

The temperature control system of vacuum annealing furnace generally uses the traditional PID control method. In recent years, many high-temperature processing furnaces have begun to add fuzzy control rules to better improve equipment performance and reduce energy consumption. Gao Xiaodong for large-scale pre-oxidation furnace nonlinear, large hysteresis and high control accuracy requirements of the characteristics of the fuzzy adaptive PID control algorithm to replace the traditional PID control method for the pre-oxidation furnace temperature control, and in the MATLAB platform simulation study, the results show that the algorithm temperature control is accurate, anti-interference ability [12]. By analyzing the characteristics of PID algorithm, Xiong Changjiong et al. designed an incomplete differential fuzzy adaptive PID control algorithm for furnace temperature control, which has the advantages of small overshooting, fast response speed and small steady state error compared with the traditional PID control [13]. The use of fuzzy control to optimize the traditional PID, without the need for an accurate mathematical model, and good robustness and small overshoot. However, the fuzzy control itself also has certain limitations, in the fuzzy adaptive PID algorithm, mainly based on the existing experience and related theories to formulate the fuzzy control rules of kp, ki and kd, the formulation of the rules has a greater impact on the control effect. At the same time, the formulation of the fuzzy rule table has a great deal of subjectivity, based on the accumulation of the subjective experience of the previous project formed by the control results of the output is not necessarily the best, so it is difficult to ensure that the control effect is optimal [14–17]. To address this shortcoming, the authors propose a fuzzy PID vacuum annealing furnace temperature control system design based on GA optimization. The system starts from the production equipment and main technical parameters, adopts advanced control algorithms, and reduces the temperature control error by optimizing the temperature control parameters of the annealing furnace. This not only saves energy, shortens the production cycle of the product, reduces the labor intensity of workers, but also mainly improves the quality of rare metal materials.

In recent years, although the economy has developed rapidly and people’s living standards have improved, environmental pollution has also received increasing attention from the public, and air pollution and water pollution are urgent problems to be solved [18–20]. In the vacuum annealing process, reasonable and effective control of heating temperature and vacuum degree can be achieved to reduce system energy consumption, reduce pollution, and improve the ecological environment [21]. So studying temperature control of vacuum annealing can not only improve economic benefits, but also promote the development of environmental protection in the industrial field, which is of great significance for building environmental civilization and harmony.

## 2. Overall design of temperature control system for vacuum return furnace

### 2.1 Hardware structure framework

**Fig 1:**
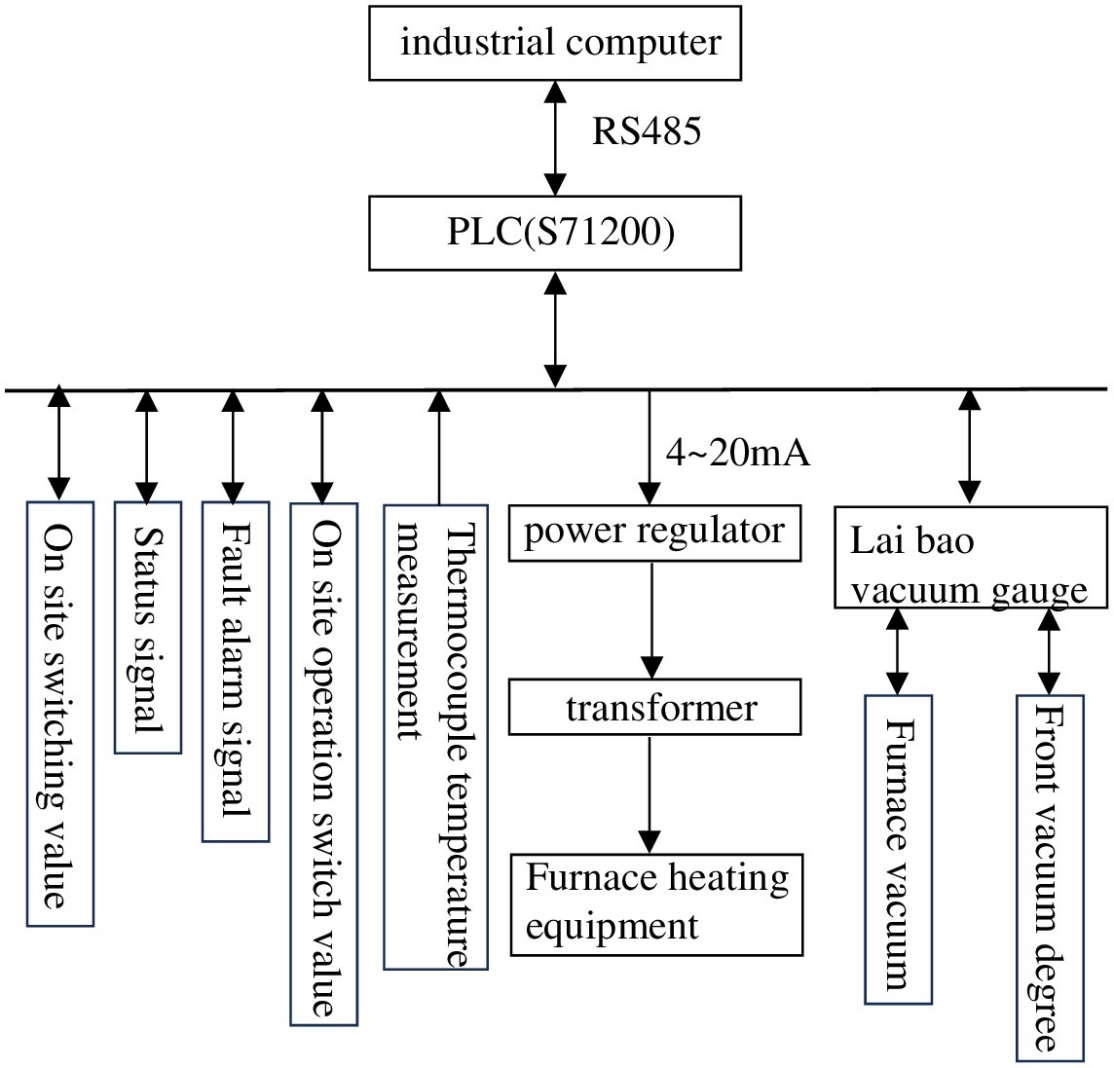
Hardware system structure diagram.

The hardware composition of vacuum annealing furnace mainly includes upper computer, PLC, and power regulator. The hardware diagram of the system is shown in Fig 1. The experiment selected Siemens S7–1200 series PLC as the main controller of the temperature control system, and the specific module selection is as follows:

(1) The CPU module selects the 1217C module, which is the backbone of the PLC and has an RS422/485 interface that supports MODBUS communication protocol [22].
(2) The digital input module adopts SM321 (DI16 × DC24V module, which is a 16 channel digital input module mainly used for on-site fault alarm and emergency stop switch signal input.
(3) The digital output module uses SM322 (DO16 × DC24V module, mainly used to control the start and stop of on-site pump valves.
(4) The analog input module uses SM331 (AI8 × 13 bit), 8-channel 8-point analog input module, mainly used for collecting and processing thermocouple signals in various temperature zones, as well as on-site pressure, flow and other signal detection.
(5) The analog output module adopts SM332 (AO4 × 12 bit), this module is a 4-channel analog output module mainly used for controlling on-site power regulation equipment, etc.
(6) The heating environment of the vacuum annealing furnace is complex, with a maximum heating temperature of 1200°C and a working temperature of 600°C~1000°C. Therefore, K-type thermocouples with strong oxidation resistance are selected as temperature measuring elements for the vacuum annealing furnace according to needs. The measurement range of K-type thermocouple is between 0°C~1300°C, with a diameter of generally 1.2 4.0mm [23]. It can measure the temperature of solid surfaces, liquids, gases, etc. in actual production. Due to its good linearity, high stability, strong oxidation resistance, and low cost, K-type thermocouples are widely used in vacuum heat treatment processes.

### 2.2 Process of temperature control system for vacuum annealing furnace

**Fig 2:**
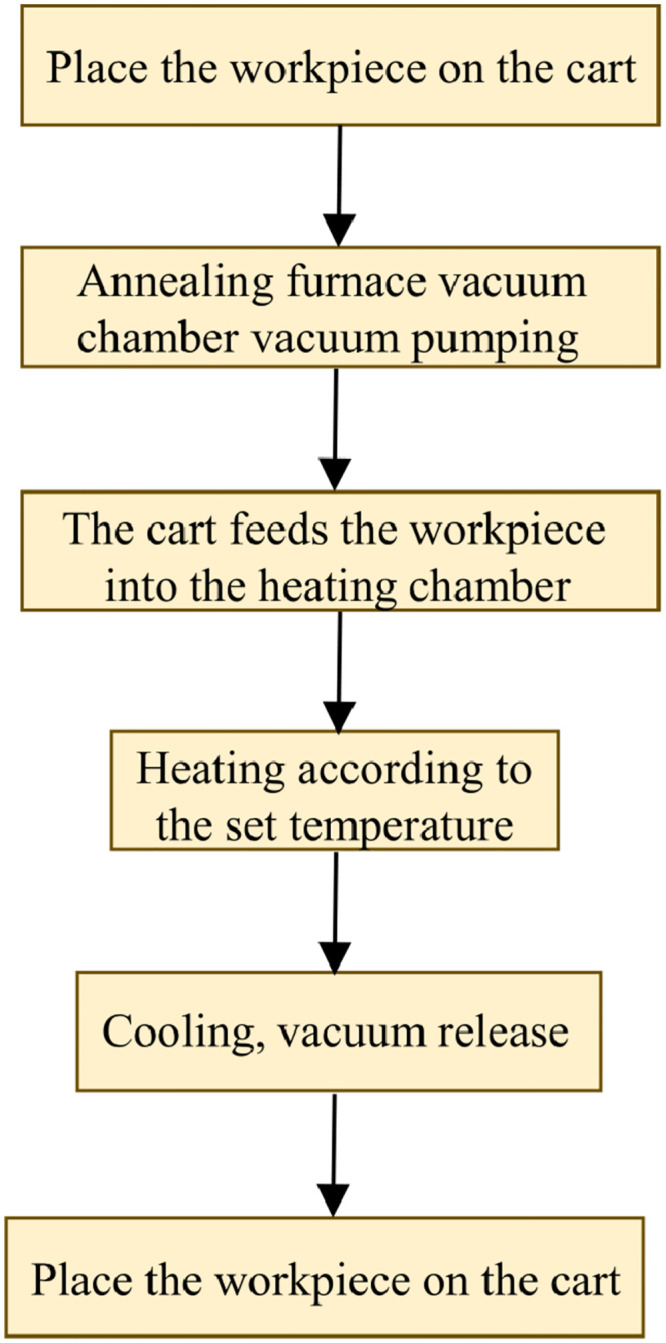
Annealing process diagram of vacuum annealing furnace.

The annealing process of the vacuum annealing furnace temperature control system is shown in Fig 2. It is preferred to place the workpiece to be annealed on the cart, close the furnace door, vacuum the vacuum annealing furnace, and then push the workpiece into the heating chamber. Then, heat and cool according to the set temperature curve. After completion, open the furnace door to release the vacuum and discharge the material. The main process parameters of the vacuum annealing furnace are shown in Table 1:

**Table 1:** Vacuum annealing furnace process parameters table.

Process parameters	Parameter ranges
Heating power	470W
Maximum heating temperature	1600°C
Rated operating temperature range	850°C~1400°C
furnace temperature uniformity	±5°C
Control accuracy	±3°C
Working vacuum	8 × 10–3Pa
boost rate	6.7Pa/h

### 2.3 Vacuum annealing furnace heating system principle analysis

**Fig 3:**
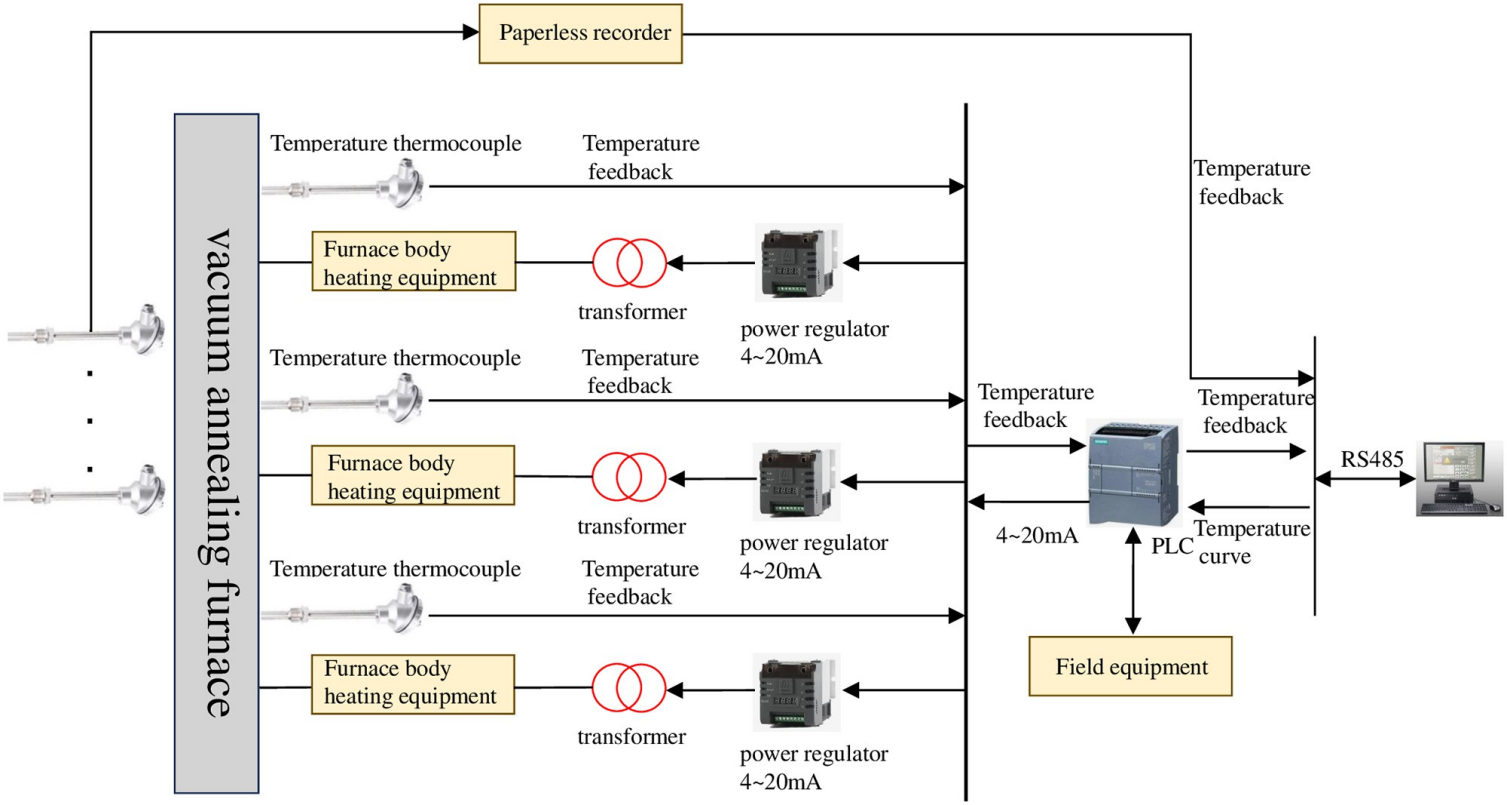
Vacuum annealing furnace heating system control schematic diagram.

The hardware composition of vacuum annealing furnace mainly includes upper computer, PLC, and power regulator. The hardware diagram of the system is shown in Fig 3. The experiment selected Siemens S7–1200 series PLC as the main controller of the temperature control system, and the heating control process is as follows:

According to the requirements of vacuum heat treatment process, the system draws the set temperature process curve through the upper computer monitoring software. By using the RS485 serial port and MODBUS communication protocol, the temperature curve is sent to the programmable controller PLC. When the vacuum degree in the annealing furnace meets the heating conditions, the PLC begins to control the heating of the annealing furnace. During the annealing process, the working temperature is generally between 600~1000°C. The PLC can receive the measured temperature values collected by thermocouples, and write the measured temperature values and set values into the upper computer software through the RS485 bus. The temperature measured values and set values are written into the temperature control strategy software through the upper computer software. The PID controller optimized by genetic algorithm completes the tuning of PID parameters and sends the control output to the PLC. The power regulator receives a simulated control signal of 4–20MA output from the PLC, and outputs a stable variable period switching value through the periodic switch, thereby controlling the time ratio of the thyristor on/off to control the transformer. Ultimately, it achieves effective control of the heating power of the system, and dynamically controls the temperature of each temperature zone of the annealing furnace, enabling the annealing temperature to accurately track the set temperature process curve.

## 3. Design of temperature control system based on GA-Fuzzy optimization

### 3.1 Vacuum annealing furnace temperature control modelling

The object of this article is a vertical structure internal heating vacuum annealing furnace. Control thermocouples and monitoring thermocouples are installed in the three temperature zones of the furnace to detect the temperature of each temperature zone. Nine workpiece thermocouples are installed around the material rack to collect the effective working area temperature in the furnace, which is used to detect the temperature uniformity inside the furnace [24]. According to the laws of thermodynamics, there are three modes of heat conduction: conduction, convection, and radiation. Due to the complex internal structure of vacuum annealing furnaces, the heating process is influenced by multiple factors, resulting in the simultaneous existence of the three modes of heat conduction. During the working process of a vacuum annealing furnace, the heat generated by the heater inside the furnace is mainly transmitted to the workpiece through thermal radiation, while inside the workpiece, the workpiece is evenly heated through thermal conduction [25]. A portion of the heat is also transmitted to the outside through the furnace wall through thermal conduction. Meanwhile, due to the weak gas flow in the heating chamber, there is also weak convective heat transfer in the heating chamber. Calculate the thermal equilibrium equation through thermodynamic law analysis:

$$\begin{array}{c} Q_{\text{total}}=Qeffective+Qloss+Qstorage \end{array}$$ (1)

In the equation:where Qtotal is the total heat generated by the annealing furnace heater. Qeffective is effective heat consumption, that is, the heat consumed by heating the workpiece. Qloss is reactive heat loss. Qstorage is the heat lost by the furnace’s heat storage [26].

(1) Calculation of effective heat consumption:

$$\begin{array}{c} Q_{\text{effective}}=M_{\text{workpieces}}C_{\text{workpieces}}\frac{dT}{dt}+M_{\text{clamp}}C_{\text{clamp}}\frac{dT}{dt} \end{array}$$ (2)

where Cworkpieces, Cclamp are the specific heat capacities of workpieces and fixtures.

(2) Calculation of reactive heat loss:

$$\begin{array}{c} Q_{\text{loss}}=Qa+Qb+Qc \end{array}$$ (3)

Where Qa is the heat loss caused by heat transfer to the furnace wall through the insulation, Qb is the heat loss caused by thermal short-circuiting, and Qc is the other heat loss, which mainly refers to the heat exported from thermocouples and vacuum tubes, as well as the dissipation of heat from the observation holes [27].

Qa refers to the heat loss caused by the heat transfer to the furnace wall through the insulation, which should include: the heat loss of the top, side and bottom parts. To wit:

$$\begin{array}{c} Q_{a}=Q_{flanking\;loss}+Q_{\text{top}\;\text{loss}}+Q_{\text{bottom}\;\text{loss}} \end{array}$$ (4)

According to the laws of thermodynamics, it can be concluded that:

$$\begin{array}{c} Q_{\text{flanking}\;\text{loss}\;}=\frac{2\pi \lambda L(T_{f}-T_{o})}{\text{ln}\;\frac{D_{2}}{D_{1}}} \end{array}$$ (5)

Where λ is the thermal conductivity of the annealing furnace insulation, L is the height of the vacuum annealing furnace, Tf and T0 are the internal and external temperatures of the annealing furnace insulation, and D1 and D2 are the internal and external diameters of the annealing furnace insulation, respectively [28].

The same reasoning leads to:

$$\begin{array}{c} Q_{bottom\;\text{loss}\;}=Q_{top\;\text{loss}\;}=\frac{T_{f}-T_{o}}{\frac{d}{\lambda F}} \end{array}$$ (6)

where Tf and T0 are the internal and external temperatures of the annealing furnace insulation, d is the thickness of the top and bottom of the annealing furnace, λ is the thermal conductivity of the annealing furnace insulation, and F is the average area of the top and bottom of the annealing furnace.

The two parts of heat loss, Qb and Qc, are difficult to calculate specifically and are usually taken based on practical experience:

$$\begin{array}{c} Q_{b}=\eta_{1}Q_{a}=\left(\%5∼\%10\right)Q_{a} \end{array}$$ (7)

$$\begin{array}{c} Q_{c}=\eta_{2}Q_{a}=\left(\%3∼\%5\right)Q_{a} \end{array}$$ (8)

(3) The heat consumed in storing heat in the furnace during the heating process is:

$$\begin{array}{c} Q_{\text{storage}\;}=GC_{m}\frac{dT}{dt} \end{array}$$ (9)

where G is the mass of the annealing furnace walls and insulation and Cm is the average specific heat capacity of the annealing furnace walls and insulation.

If the voltage of the heater is U, the resistance of the heater is R, and n is the total number of heaters [29]. Then:

$$\begin{array}{c} Q_{\text{total}\;}=n\frac{U^{2}}{R} \end{array}$$ (10)

The above analysis can be introduced:

$$n\frac{U^{2}}{R}=M_{G}C_{G}\frac{dT}{dt}+M_{J}C_{J}\frac{dT}{dt}+[\frac{2\pi \lambda L(T_{f}-T_{o})}{\text{ln}\;\frac{D_{2}}{D_{1}}}+\frac{2(T_{f}-T_{o})}{\frac{d}{\lambda F}}](1+\eta_{1}+\eta_{2})+GC_{m}\frac{dT}{dt}$$ (11)

When T0≪Tf

$$\begin{array}{c} C^{*}=\frac{1}{2\lambda [\frac{\pi L}{\left(\text{ln}D_{1}/D_{2}\right)}+\frac{F}{d}](1+\eta_{1}+\eta_{2})} \end{array}$$ (12)

This is obtained by bringing Eq (12) into the collation:

$$\begin{array}{c} n\frac{U^{2}}{R}=(M_{G}C_{G}+M_{J}C_{J}+GC_{m})\frac{dT}{dt}+\frac{T_{f}}{C^{*}} \end{array}$$ (13)

When both the input and output quantities have corresponding increments, simplifying the above Eq (13) yields an equation reflecting the relationship between the increments as follows:

$$\begin{array}{c} \tau \frac{d\Delta T}{dt}+\Delta T=K\Delta U \end{array}$$ (14)

$$\begin{array}{c} \tau =C^{*}(M_{G}C_{G}+M_{J}C_{J}+GC_{m}) \end{array}$$ (15)

$$\begin{array}{c} K=\frac{2nu_{0}}{R}C^{*} \end{array}$$ (16)

The above equation is obtained by Laplace transform:

$$\begin{array}{c} G\left(s\right)=\frac{T(s)}{U(s)}=\frac{K}{1+Ts} \end{array}$$ (17)

Considering the delay nature of the system, the final transfer function of the system is:

$$\begin{array}{c} G\left(s\right)=\frac{K}{1+Ts}e^{-\tau s} \end{array}$$ (18)

System identification is to analyze and process the experimental or actual operation data of the system, so as to get the information reflecting the main operation characteristics of the system, and establish the mathematical model of the controlled object according to the obtained information. Vacuum annealing furnace temperature control system of the intrinsic change mechanism is complex and variable, it is difficult to use conventional mathematical reasoning methods to establish a suitable mathematical model, so the following considerations of the system identification method to obtain the mathematical model.

**Fig 4:**
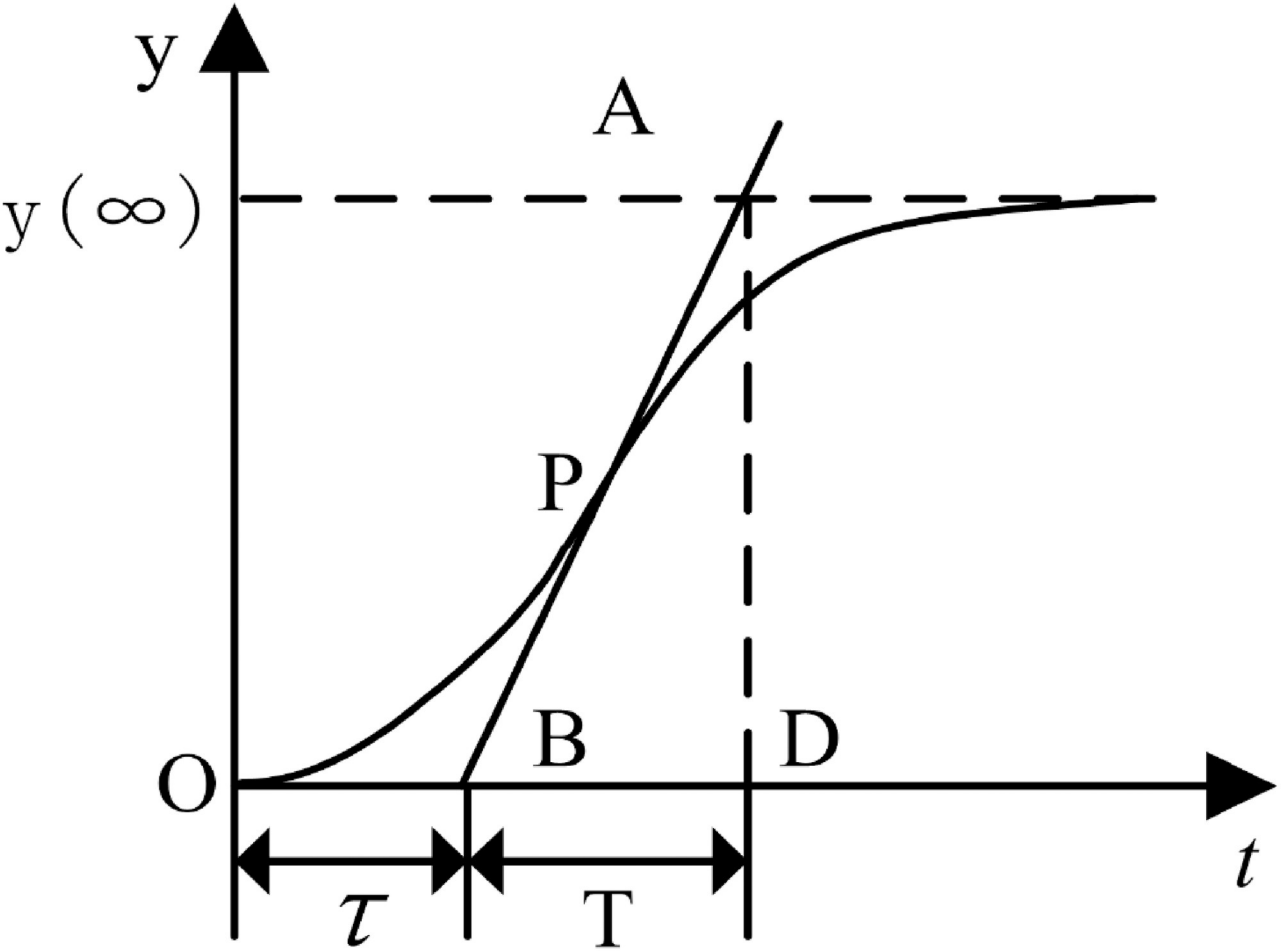
Step response curve of controlled object.

**Fig 5:**
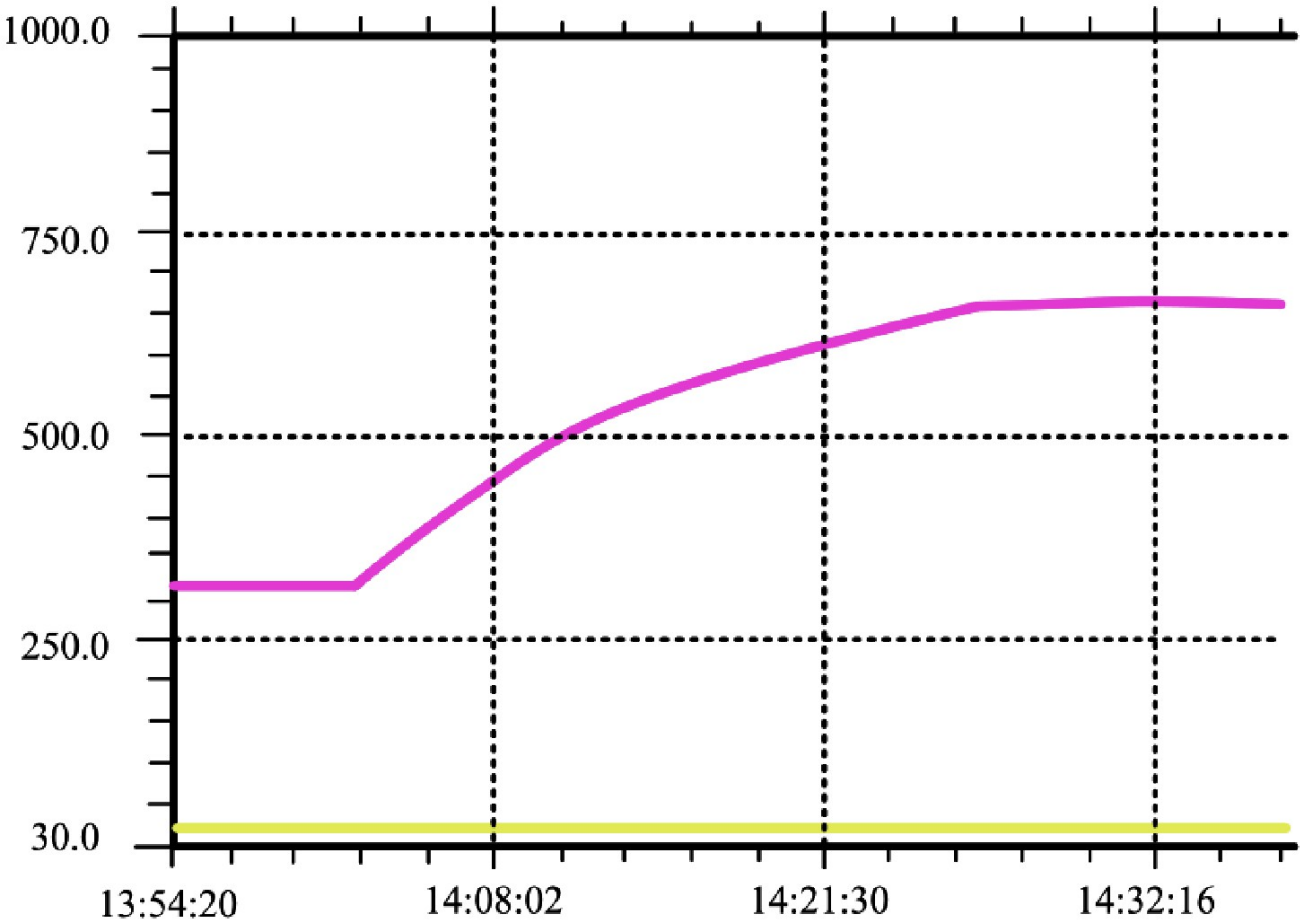
Step response curve of vacuum annealing furnace.

There are many classic system identification methods. Compared to other identification methods, the step response method has a clear physical meaning of parameters and is easy to implement in industrial fields. Therefore, this article uses the step response method to identify the temperature control system model. If the temperature control response curve of the controlled object is close to an S-shaped curve, as shown in Fig 4, the parameters of the response curve can be obtained using the tangent method. In the figure, P is the inflection point of the step response curve. Make a tangent with the highest slope through point P, intersect with the time axis at point B, and intersect with the steady-state asymptote of the curve at point A.T and *τ* are determined from the time values at points A and B. If u(t) and Δu(t) denote the step input and the input variation, respectively, and y(Θ) and y(∞) denote the starting and steady state values of the output y(t), respectively, then the static gain K can be calculated directly from the following equation:

After multiple experiments, the step response curve of vacuum annealing furnace temperature control is shown in Fig 5.

combined with the actual collection of data in the field, the use of tangent method of analysis and calculation, and ultimately take K = 6, T = 532, *τ* = 40. vacuum annealing furnace temperature control of a single-temperature zone of the transfer function is:

$$\begin{array}{c} G\left(s\right)=\frac{6}{1+532s}e^{-40s} \end{array}$$ (19)

### 3.2 Fuzzy PID control algorithm

In recent years, fuzzy PID control algorithms are increasingly used in the manufacturing industry, most of which are used to improve the accuracy of the equipment, reduce the response time and reduce overshoot, etc., with the traditional sense of the PID control method compared to the fuzzy PID can be better suited to contain disturbances in the workshop environment, which is combined with the actual processing, the development of fuzzy rules in the empirical sense of the anti-jamming ability of the equipment is better in this regard for the production and processing of the manufacturing enterprise has a “quality” of improvement.

(1)Fuzzy Controller By comparing the temperature data collected by thermocouples with the temperature values set by the annealing furnace, the error (e) and error rate of change (ec) can be obtained [30]. The error (e) and error rate of change (ec) can be calculated as input variables for the fuzzy controller. After fuzzification, fuzzy reasoning, and deblurring, ΔKp, ΔKi, and ΔKd can be obtained, and then the fuzzy PID parameters can be obtained:

$$\begin{array}{c} K_{p}={\ddot{K}}_{p}+\Delta K_{p} \end{array}$$ (20)

$$\begin{array}{c} K_{i}={\ddot{K}}_{i}+\Delta K_{i} \end{array}$$ (21)

$$\begin{array}{c} K_{d}={\ddot{K}}_{d}+\Delta K_{d} \end{array}$$ (22)

**Fig 6:**
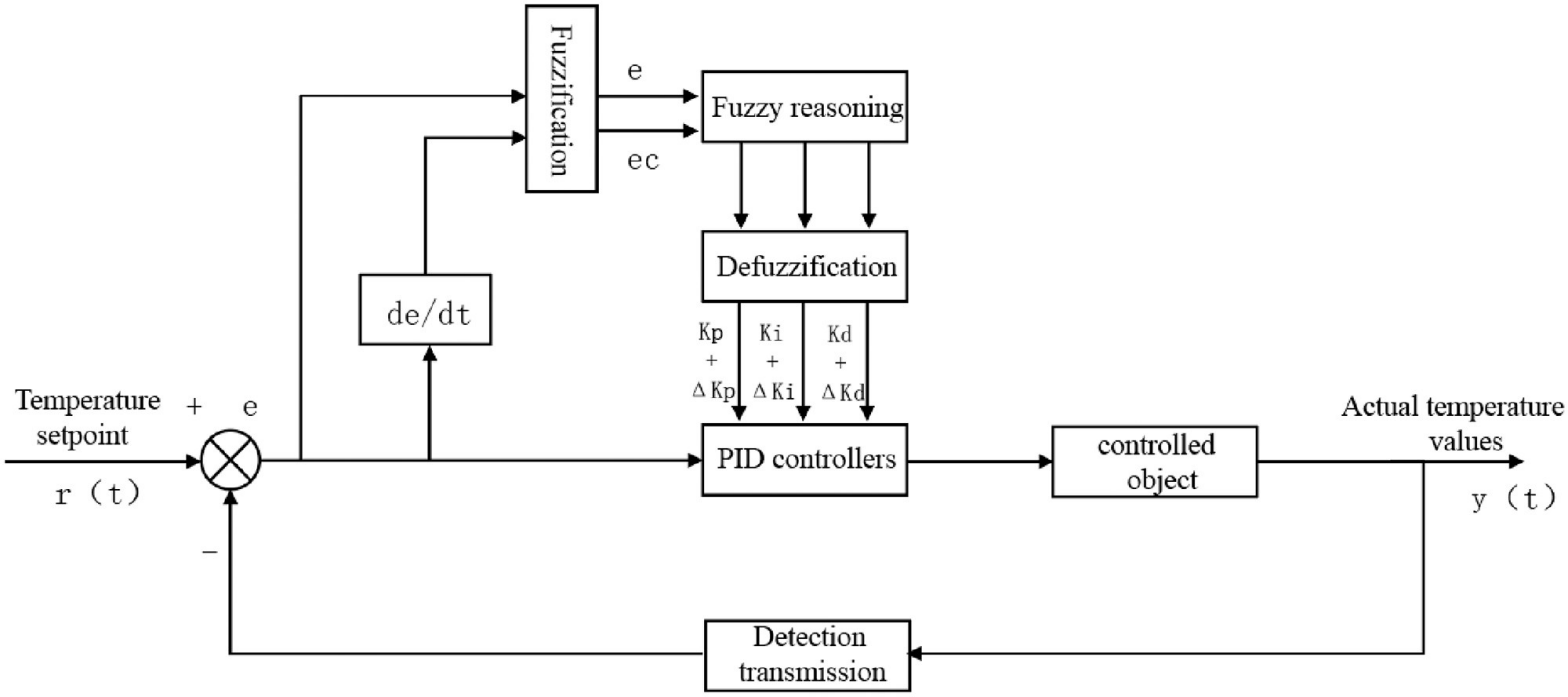
Fuzzy PID control structure diagram.

In Eqs (20), (21), and (22), ${\ddot{K}}_{p}$. ${\ddot{K}}_{i}$. ${\ddot{K}}_{d}$ is the basic value set by the PID controller to be adjusted, in the formula ΔKp. ΔKi. ΔKd is Kp. Ki. Kd Continuously revise the values to complete the fuzzy optimization control of fixed parameter PID. Fig 6 is the structure diagram of fuzzy PID control.

(2)Fuzzy distribution of input and output quantities

The input and output domains of the fuzzy controller are set to [-6, 6], [-3, 3], respectively. The temperature errors (e) and (ec) of the vacuum annealing furnace are used as the controller inputs, and ΔKp, ΔKi, and ΔKd are used as the controller outputs. To ensure the accuracy of the simulation, the Gaussian function is fused with the trigonometric function, and the center of gravity method is used to solve the fuzzy calculation.

In the temperature control system of vacuum annealing furnace, the annealing furnace temperature is continuously detected by thermocouples, and the relationship between temperature error e and error change rate ec is calculated. Then, the fuzzy PID parameters are adjusted online using a PID controller to obtain the ideal effect of vacuum annealing furnace temperature control.

In fuzzy controllers, the control rule table is closely related to the control effect. The range of changes in input e (t) and ec (t) can be divided into seven cases: negative large (NB), negative medium (NM), negative small (NS), zero (Z), positive small (PS), positive small (PM), and positive large (PB). The free combination of e (t) and ec (t) can obtain 49 rules, and each rule provides fuzzy quantities for the three parameters kp, ki, and kd, including 8 cases: negative large (NB), negative medium (NM), negative small (NS), negative zero (NZ), positive zero (PZ), and positive small (PS).) Center (PM) and Large (PB) [31]. This article takes the following rules as an example.

If e(t) is NS and ec(t) is Z, then kp is PZ and ki is PZ and kd is PS.

### 3.3 Fuzzy PID controller optimized based on genetic algorithm

Genetic algorithm (GA) is a parallel random search optimization method formed by simulating natural genetic mechanisms and biological evolution theory. It provides an effective approach to approaching the optimal solution for some complex and cumbersome solution spaces, so it is widely used in industrial processing processes. Due to the lack of theoretical basis and subjective initiative in the formulation of fuzzy rules for vacuum annealing furnace controllers, fuzzy rules may not necessarily be the optimal and ideal type.

**Fig 7:**
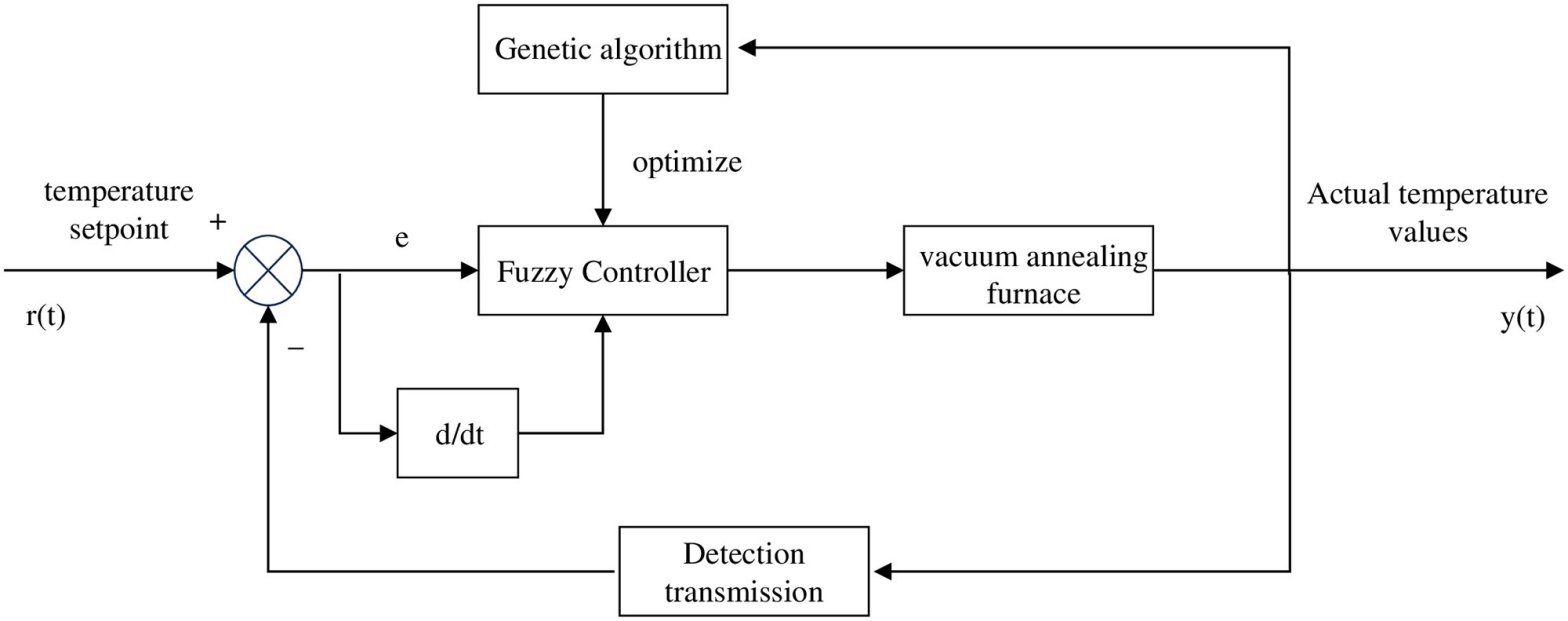
Structure diagram of genetic algorithm optimized fuzzy controller.

This experimental project is completed offline optimization, due to the high temperature characteristics of the vacuum annealing furnace and the site environment is more complex characteristics, there are some difficulties in online optimization, genetic algorithm optimization of fuzzy controllers is relatively complex, nowadays optimization of fuzzy controllers are completed offline; that is, now through the simulation of the study achieved satisfactory results in the implantation of the actual fuzzy controllers, genetic algorithm optimization of the fuzzy controller The schematic diagram is shown in Fig 7.

**Fig 8:**
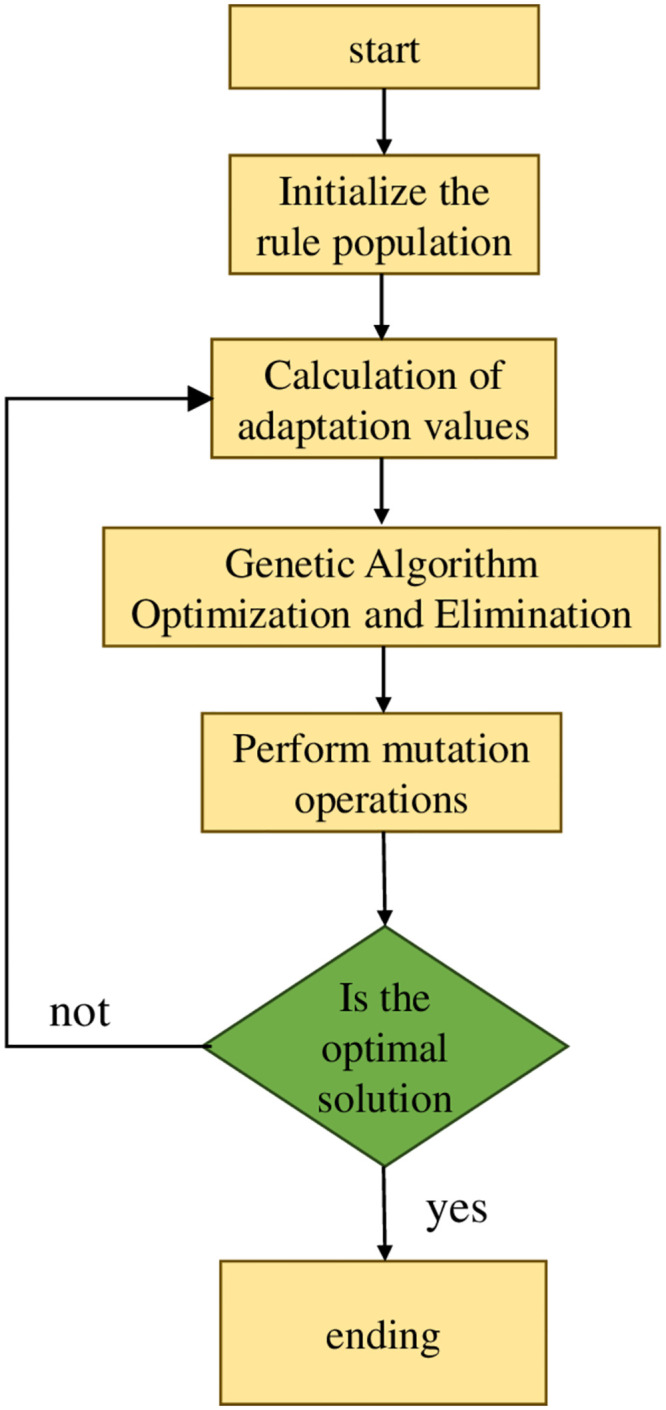
Genetic algorithm flowchart.

The main steps of genetic algorithm include: encoding the target parameters; Population initialization; Calculate the fitness of each individual in the population separately; Determine the individuals retained and eliminated in the population; Perform crossover operations based on crossover probability; Perform mutation operations based on mutation probability. After completing the target parameter encoding and population initialization, the operation is repeated until the individual with the highest fitness is selected. After decoding, the optimal solution for the target is obtained. The flowchart of the genetic algorithm is shown in Fig 8.

Generally the selection of fuzzy control rules is based on the specific characteristics of the object to be compiled, combined with expert knowledge or the experience of the actual staff to complete, so in the design of the subjective factors play an important role, with non-objective, imperfect shortcomings. In this design, the affiliation function is coded in binary, and the control rules are coded in decimal, which is a hybrid coding method conducive to the efficient genetic operation, and using the classical mathematical coding method, the control rules are taken as a whole, and several different fuzzy control rules are taken as a group, and the genetic operation is applied to each individual, and the performance diversity index is measured at the level of the individual.

(1)Binary encoding

The parameters to be optimized for the PID of this vacuum annealing furnace are Kp, Ki, and Kd. It is known that there are 49 combinations of Kp, Ki, and Kd, with a total of 147 combinations in a group of three parameters. There are only 0 and 1 binary numbers, making the combination calculation relatively simple and convenient [32]. Therefore, the binary encoding method is chosen for the optimization of the PID parameters of the vacuum annealing furnace.

**Fig 9:**
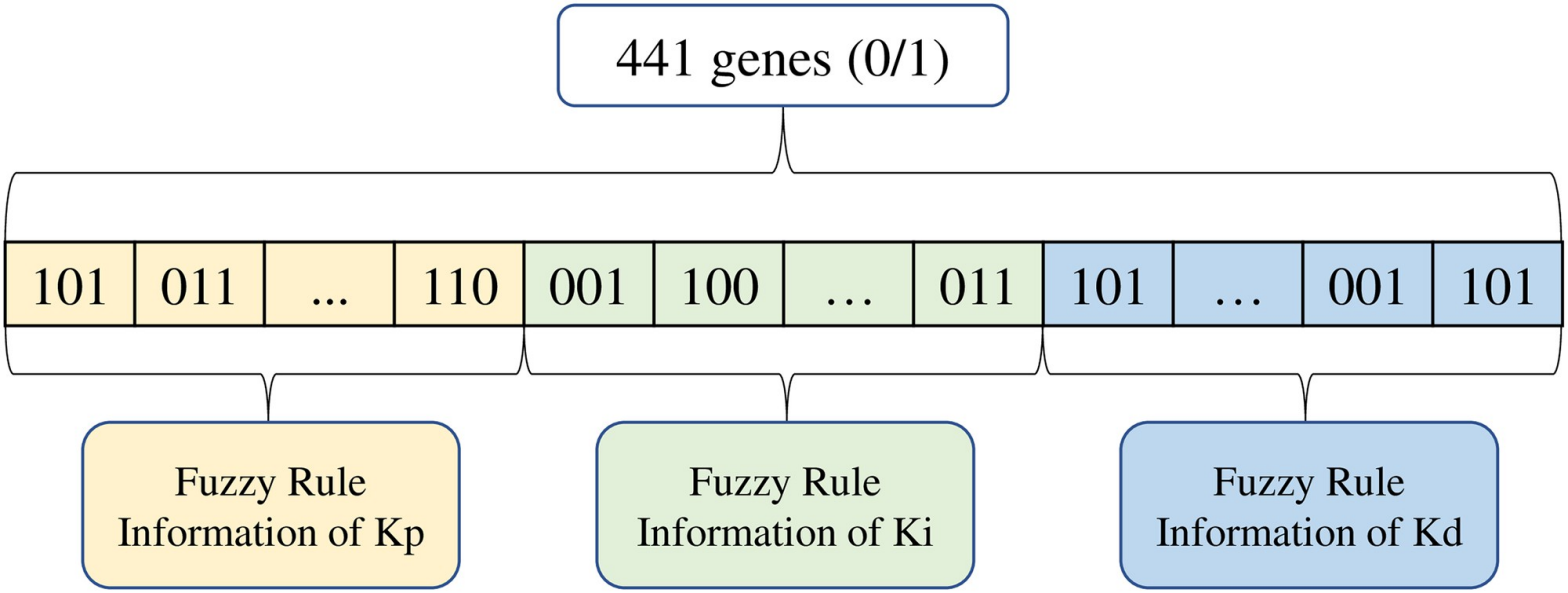
Example of individual encoding.

Select binary numbers “000” to “111” to represent decimal numbers 0, 1, 2, 3, 4, 5, 6, and 7, where these 8 numbers represent eight rules in the fuzzy subset: NB (negative major), NM (negative middle), NS (negative minor), NZ (negative zero), PZ (positive zero), PS (positive minor), PM (positive middle), PB (positive major) [33]. Therefore, there are 441 genes on one chromosome, including Kp. Ki. Kd Three parameter information. The individual binary encoding is shown in Fig 9.

**Fig 10:**
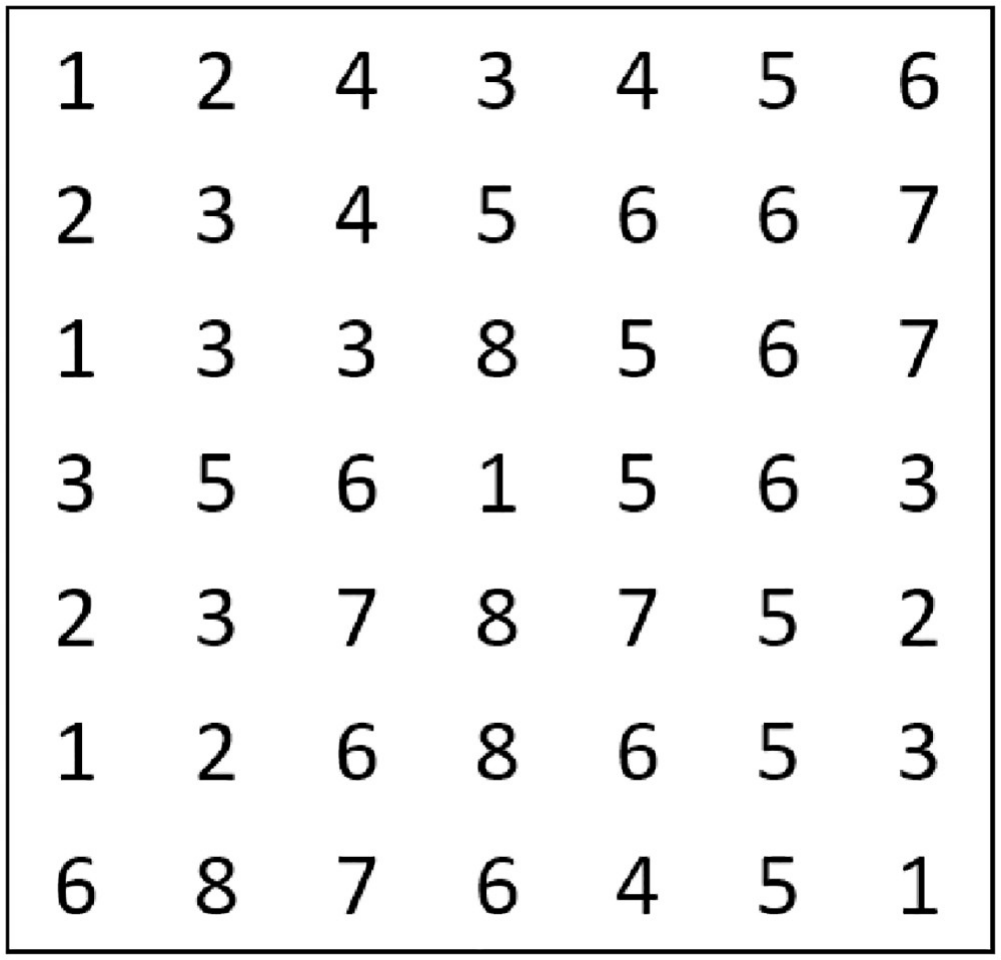
Example of Kp parameter decoding rule representation.

Through genetic algorithm for global optimization, the decoding situation is shown in Fig 10, which represents the complete rule table of a parameter (such as kp). By applying it to the fuzzy controller, it can be seen from Eqs (20), (21), and (22) that the fuzzy controller outputs the optimal parameter increment at this time, calculates the updated values of kp, ki, and kd, and then implements PID control until the iteration number ends, retaining the optimal parameter combination as the optimal solution.

(2)Fitness function

The commonly used objective function is used as the performance standard for control systems. The integral performance indicator ITAE can be used to evaluate the dynamic and static performance of control systems, such as response time, overshoot, etc. Its calculation formula is shown in Eq (23). The smaller J (ITAE), the shorter the response time t, the smaller the absolute deviation |*e*(*t*)|, and the higher the accuracy. In genetic algorithms, for ease of calculation, the objective function can be appropriately transformed to obtain the fitness function f, as shown in Eq (24). The larger the fitness value, the better the genetic optimization effect, which can determine the search direction and range based on the fitness value [34]. For the convenience of computer calculation, discretize the integral function to obtain Eq (25), where ΔT represents the sampling time.

$$\begin{array}{c} J\left(ITAE\right)=\int_{0}^{\infty }t\left|e\left(t\right)\right|dt \end{array}$$ (23)

$$\begin{array}{c} f=\frac{1}{1+J(ITAE)} \end{array}$$ (24)

$$\begin{array}{c} \Delta J=t|e(t)|\Delta T \end{array}$$ (25)

(3)Genetic Algorithm Optimization Steps

Population initialization: The initial population can be randomly generated, and in order to ensure good diversity of the population, the number of individuals is generally between 30 and 100 [35, 36]. Similarly, the choice of algebra also needs to be appropriate. If there are too few algebras, the optimal goal cannot be found; If there are too many algebras, the calculation time is too long, so the algebra is generally 30–100.

Selection: The process of eliminating the best and the worst from the current population based on certain criteria is called selection, which aims to select the best individuals so that they have a chance to reproduce their offspring as parents. There are various ways to select the selection operator, such as elite strategy, roulette algorithm or fitness value ranking, etc [37]. In order to prevent the population from maturing too early, and at the same time to improve the iteration rate, this paper adopts a combination of elite strategy and fitness value ranking selection method.

Crossover: The process of two individuals in a population genetically interchanging is called crossover, which can be classified as single-point crossover, two-point crossover, and multi-point crossover. The crossover operator Pc (also known as crossover probability) determines the ability of global optimisation, which is usually between 0.3 and 1.

Mutation: The process of changing a gene somewhere in an individual in a population is called mutation. The variation operator Pm (also known as the probability of variation) determines the ability of the algorithm to locally find an optimal solution, and is generally in the range of 0.01 to 0.1.

## 4. Simulation experiment verification

**Fig 11:**
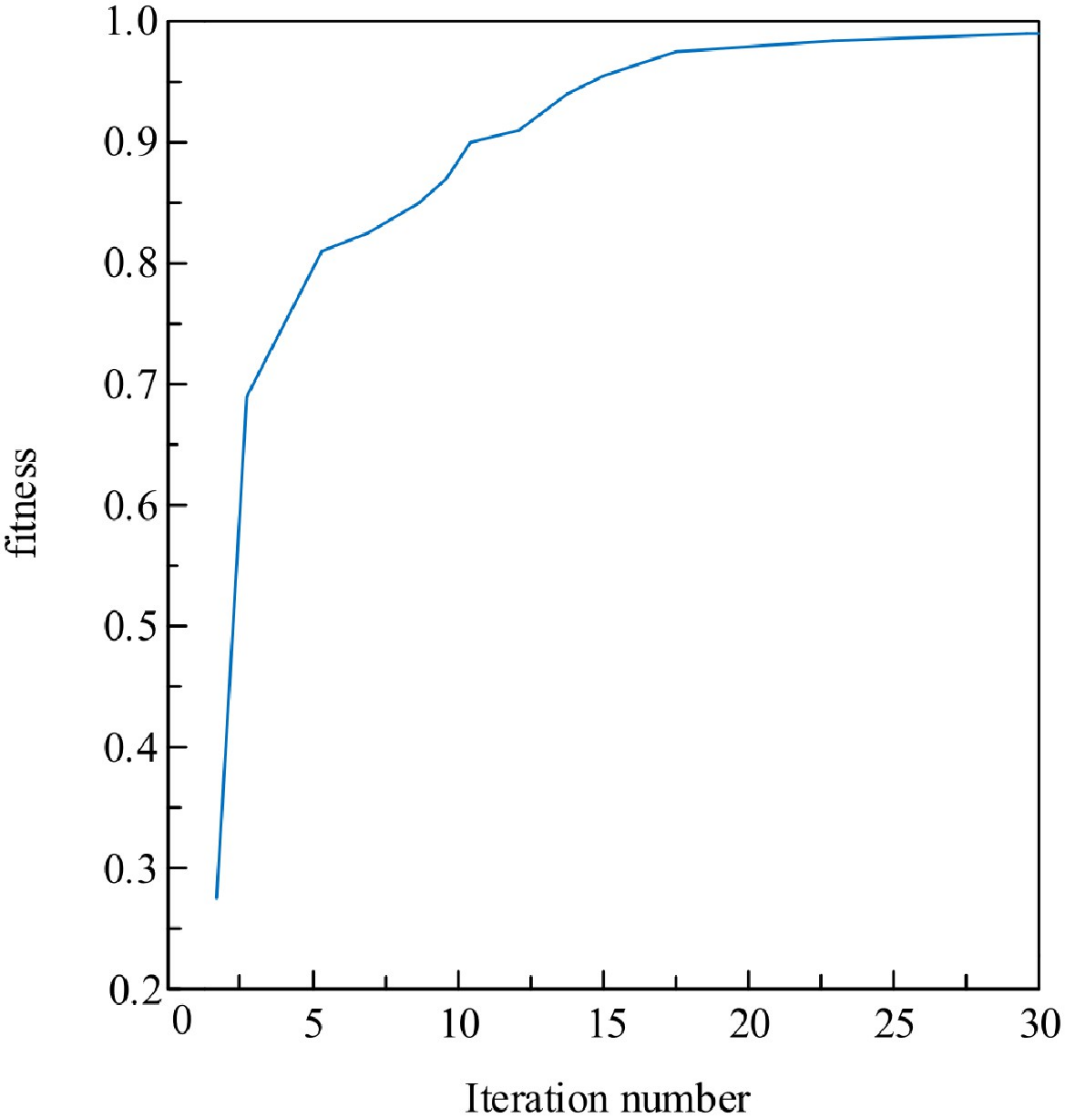
Example of Kp parameter decoding rule representation.

Set the genetic algorithm iteration number n = 30, the number of genes on each chromosome is 441, the population is set to 30, the crossover probability P1 = 0.9, and the mutation probability P2 = 0.1. Simulate using MATLAB to obtain the fitness function distribution in Fig 8 and the objective function distribution in Fig 11, respectively.

**Fig 12:**
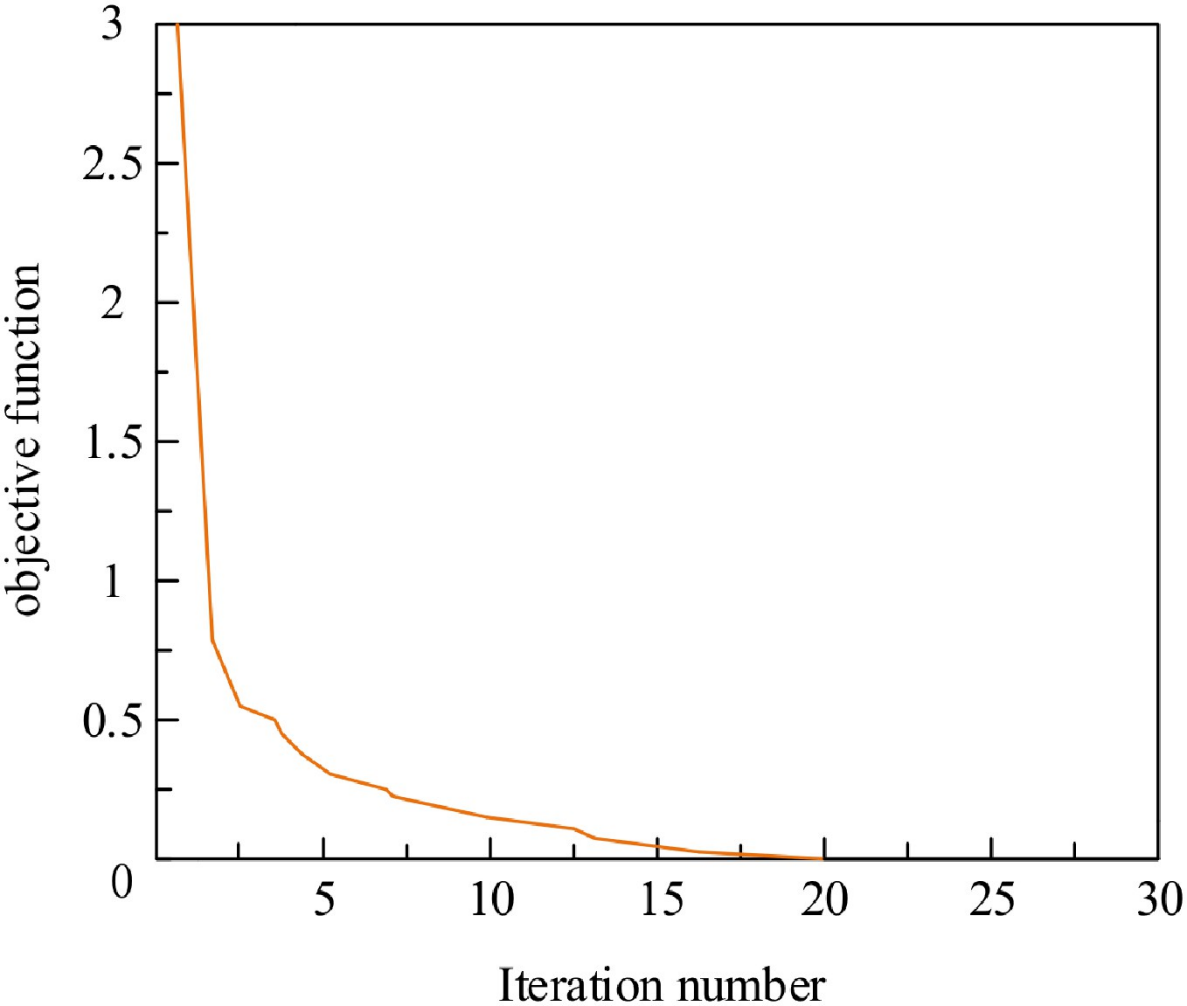
Distribution of the objective function.

By analyzing the distribution curve of the fitness function in Fig 12, it can be concluded that the overall trend of fitness gradually tends towards the numerical value 1 as the number of iterations increases. The fitness value of the curve ultimately tends towards 0.9938, which is very close to 1, indicating that it is very close to the optimal solution.

Analyzing the distribution curve of the objective function in Fig 12, it can be seen that as the number of iterations increases, the objective function curve continuously tends towards the vicinity of 0. When the number of iterations reaches 17, the vertical coordinate of the curve is basically 0, that is, the J (ITAE) integral performance indicator is 0, and the control achieves the ideal effect.

**Fig 13:**
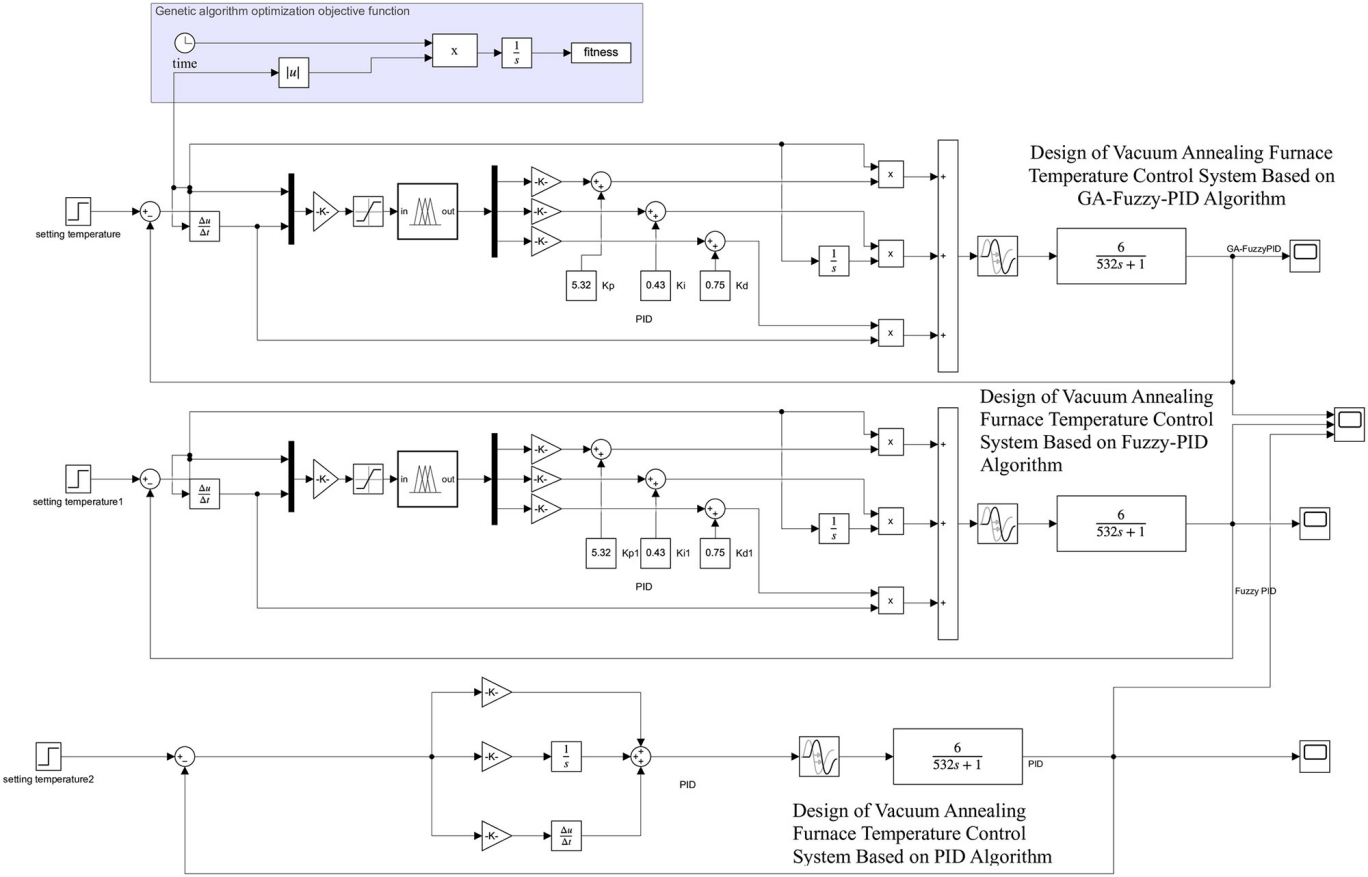
Simulation model of single temperature zone temperature control system for vacuum annealing furnace.

By decoding the above fitness functions, the optimal control rules for the three parameters Kp, Ki, and Kd of the controller are obtained. Analysis shows that when the control effect is optimal, the parameters are: Kp = 5.32, Ki = 0 43, Kd = 0 75. Finally, bring this set of parameters into the fuzzy PID controller. Through modeling and simulation using Matlab software, the simulation model diagram of the vacuum annealing furnace temperature control system under the three control modes shown in Fig 13 was obtained.

**Fig 14:**
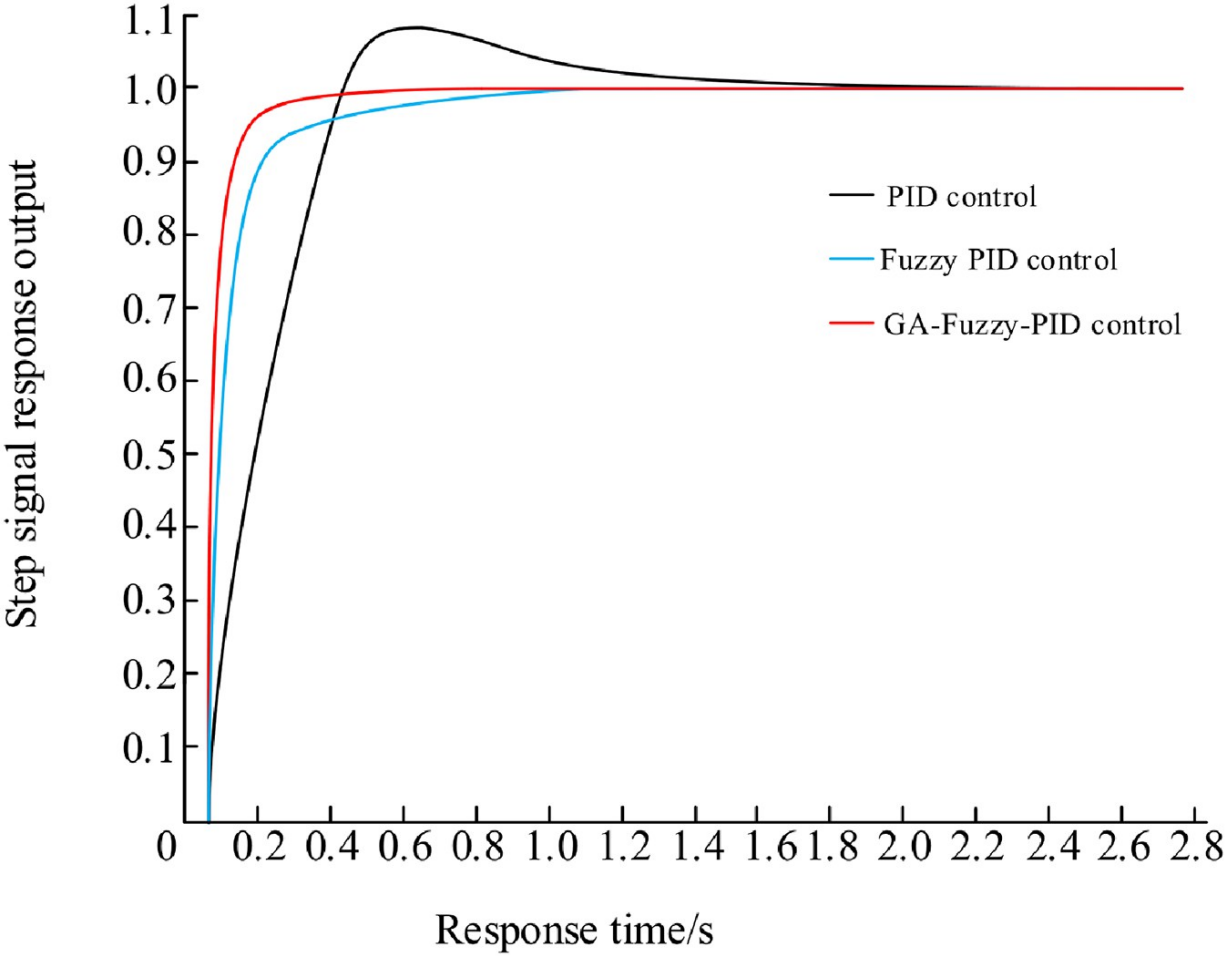
Vacuum annealing furnace temperature control system simulation waveforms.

Vacuum annealing furnace single-temperature zone temperature control system simulation process is as follows: at the beginning of t = 0 when the input unit step signal, Kp = 5.32, Ki = 0. 43, Kd = 0. 75 this group of optimal parameters into the PID controller, the simulation of the simulation to obtain the three groups of different control algorithms shown in Fig 14 under the waveform curve.

From the analysis of the waveform curves of three different control methods in Fig 14, it can be concluded that under GA fuzzy PID control, the waveform has no overshoot, and the rise time and adjustment time are better than fuzzy PID and traditional PID control methods. In order to further analyze the performance indicators of the vacuum annealing furnace temperature control system, the following waveform diagrams will be analyzed.

**Fig 15:**
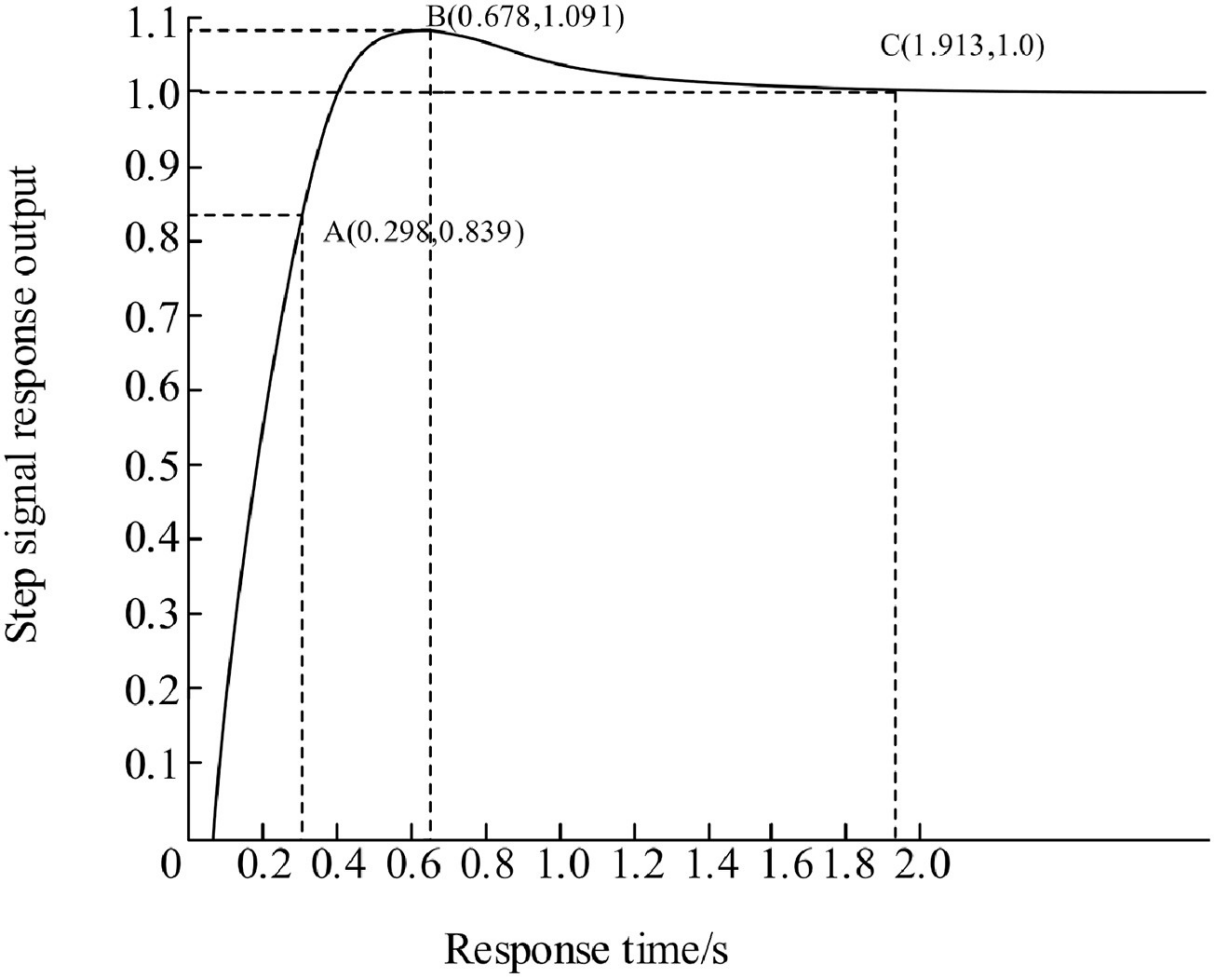
PID control simulation waveform diagram.

From the analysis of the PID control simulation waveform in Fig 15, it can be seen that the curve shows a smooth upward trend starting from point A (0.298, 0.839), reaching the highest point at point B (0.678, 1.091), and reaching a stable state at point C (1.913, 1.0). After analysis and calculation, the rise time is 0.298 seconds, the adjustment time is 1.913 seconds, and the overshoot is 9.1%.

**Fig 16:**
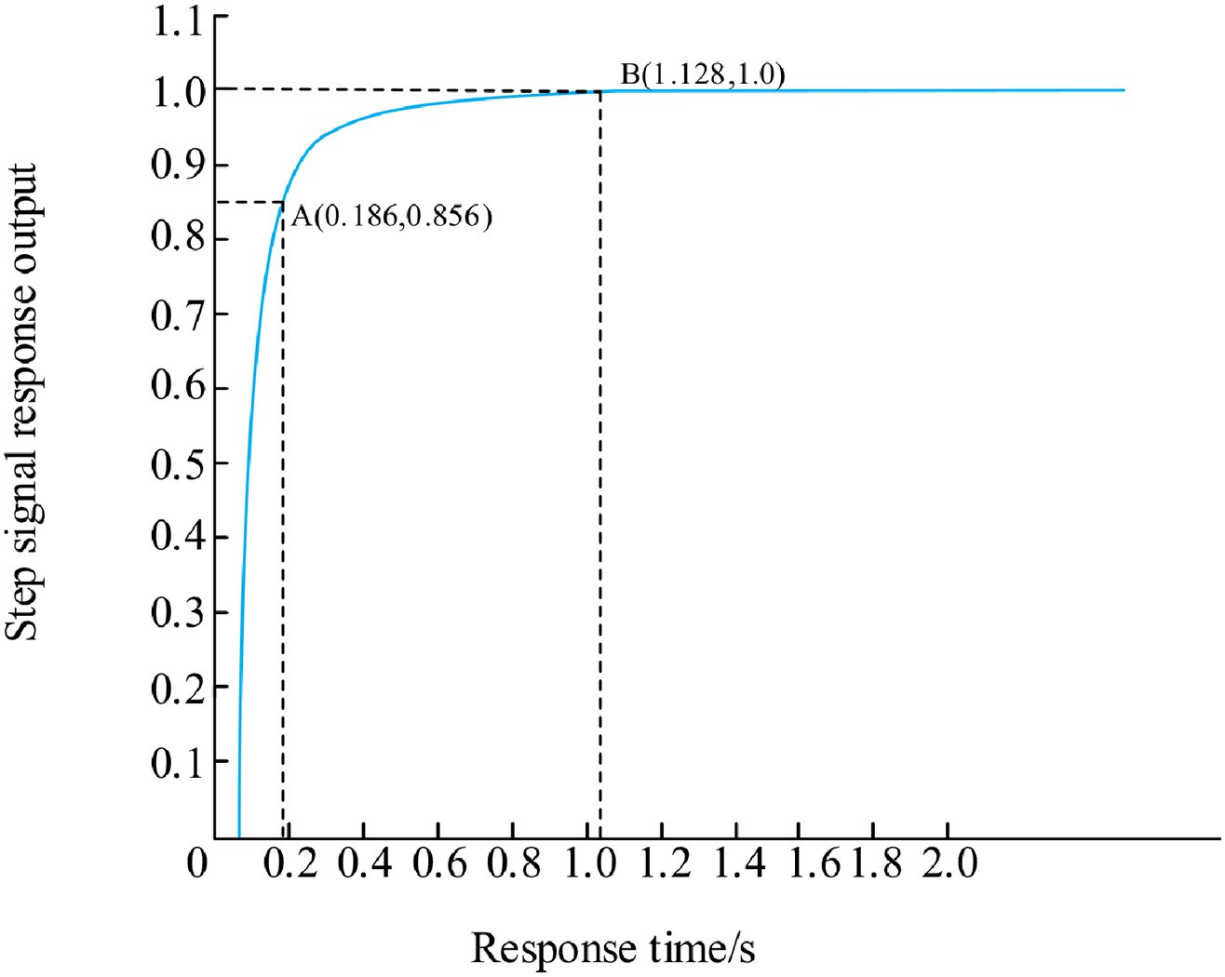
Fuzzy PID control simulation waveform diagram.

Fig 16 is the simulation waveform chart under fuzzy PID control. Through analysis, it is concluded that the coordinate curve at point A (0.186, 0.856) starts to show a smooth upward trend, and reaches a stable state at point B (1.128, 1.0) without overshoot.the rise time is 0.186 seconds, the adjustment time is 1.128 seconds, Compared with the traditional PID, the rise time is reduced by 37.5%, and the response speed is increased by 41%.

**Fig 17:**
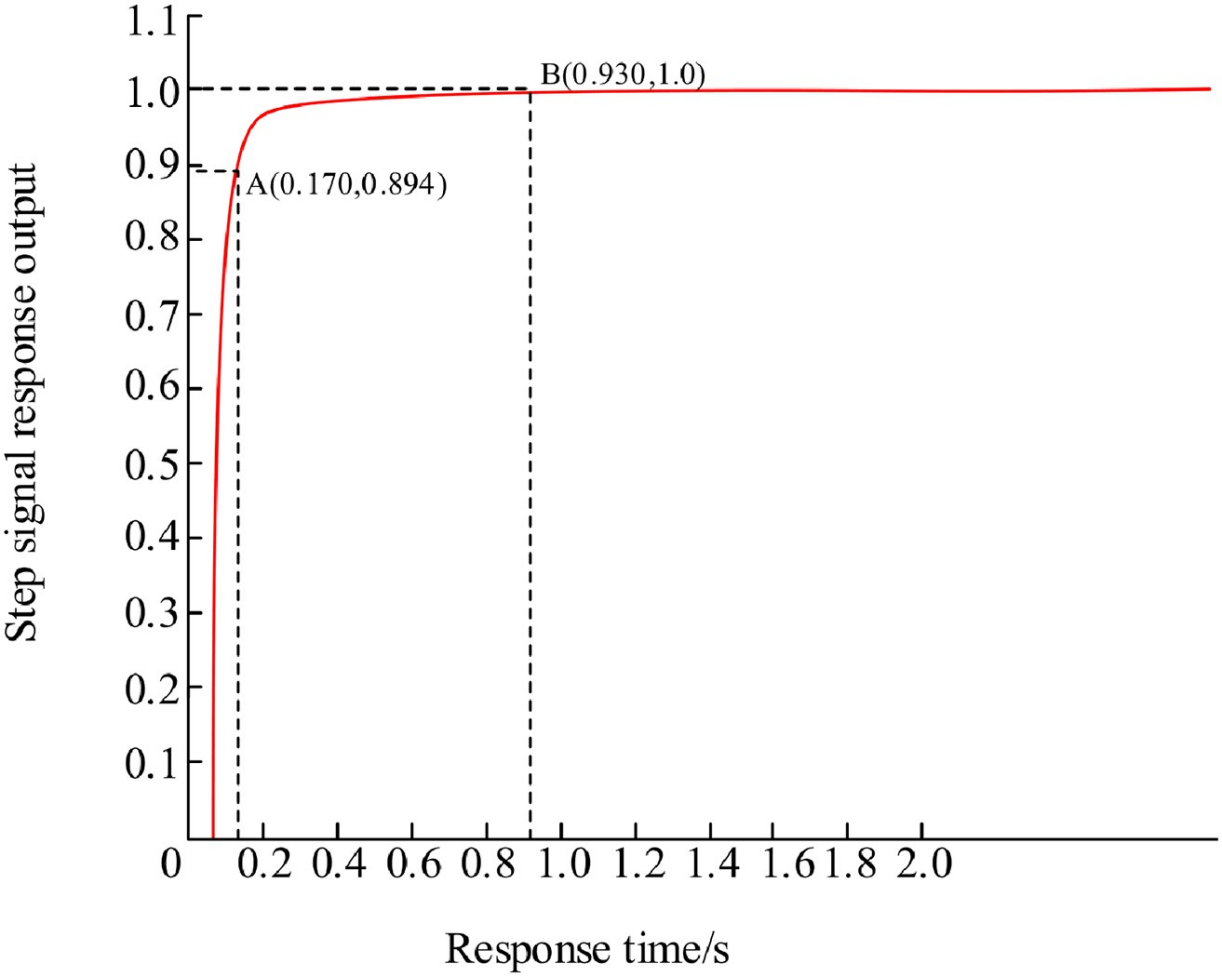
GA-Fuzzy PID control simulation waveform diagram.

Fig 17 is a simulation waveform based on GA-fuzzy PID control. Analysis shows that the coordinate curve at point A (0.170, 0.894) shows a smooth upward trend, and reaches a stable state at point B (0.930, 1.0). the rise time is 0.170 seconds, the adjustment time is 0.930 seconds, Compared with fuzzy PID, the rise time is reduced by 8.6%, and the response speed is improved by 17.6%.

In order to further compare the tuning effects of three control methods (traditional PID, fuzzy PID, and fuzzy PID based on genetic algorithm), the performance index parameters of step response were compared using rise time, adjustment time, and overshoot. The results are shown in Table 2. From the results, it can be seen that the traditional PID has a shorter rise time and adjustment time, but a larger overshoot; Compared with traditional PID, fuzzy PID greatly reduces overshoot; The fuzzy PID control method based on genetic algorithm achieves no overshoot and Compared with fuzzy PID, the rise time is reduced by 8.6%, and the response speed is improved by 17.6%.

**Table 2:** Comparison of control effects of three methods.

Control methods	rising time	Adjustment time	overshoot
Traditional PID	0.298s	1.913s	9.1%
Fuzzy PID	0.186s	1.128s	0%
GA-Fuzzy PID	0.170s	0.930s	0%

Through the comparative analysis of the above three sets of simulation waveforms, it can be concluded that the temperature control system of the vacuum annealing furnace based on the GA-fuzzy PID control algorithm has the fastest response speed and the highest control accuracy. This indicates that genetic algorithm can further improve the performance indicators of the vacuum annealing furnace temperature control system by optimizing fuzzy PID rules.

**Fig 18:**
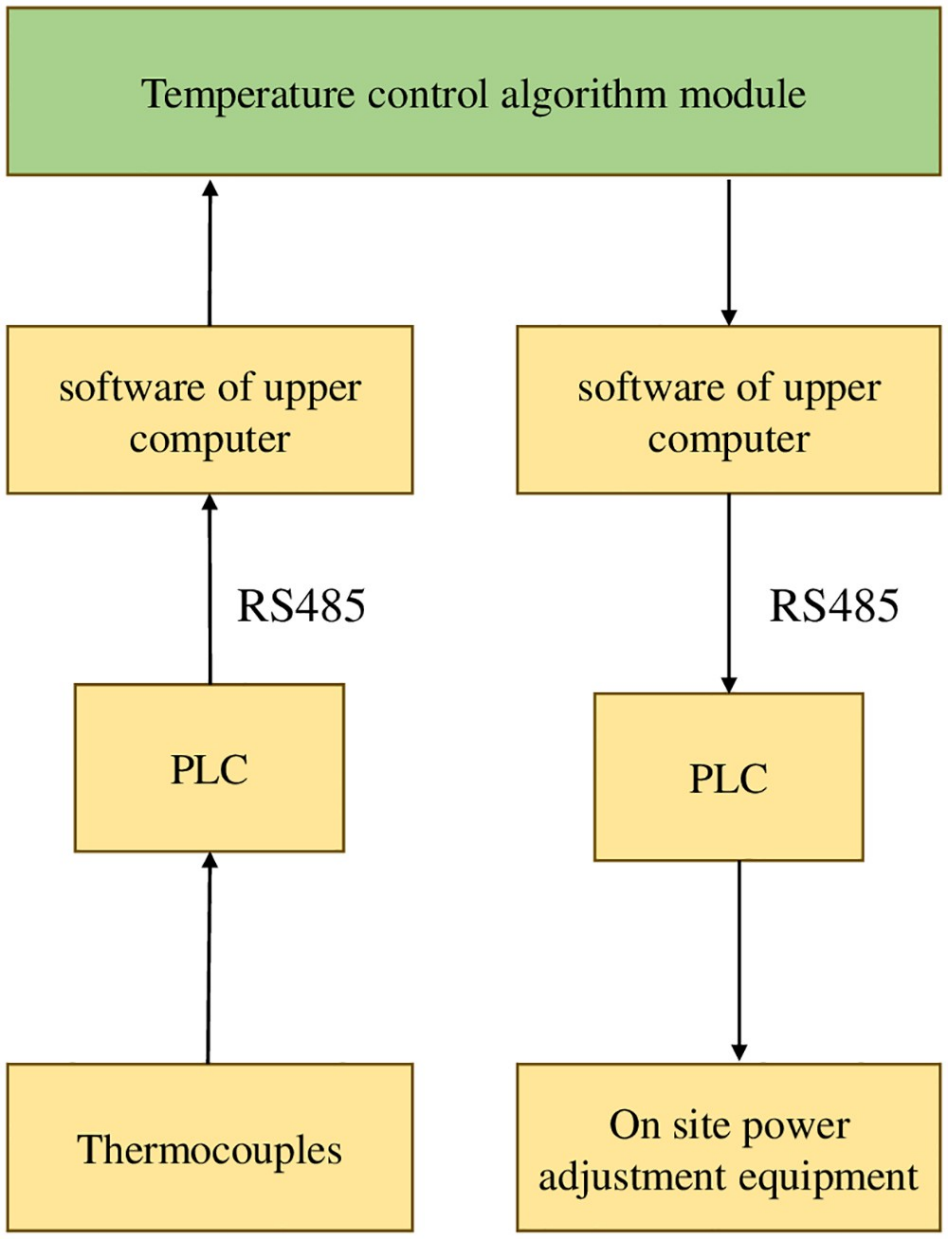
Flow chart of temperature control algorithm implementation.

During the temperature control process, the PLC transmits the measured temperature values and temperature setting values collected by the thermocouple to the upper computer software through the RS485 bus. The upper computer software calls the temperature algorithm module in Matlab, writes the measured and set temperature values into the temperature control algorithm program, reads the optimized control output, and sends the control signal to the PLC through the RS485 bus to complete the temperature control of the annealing furnace. The specific implementation process is shown in Fig 18.

**Fig 19:**
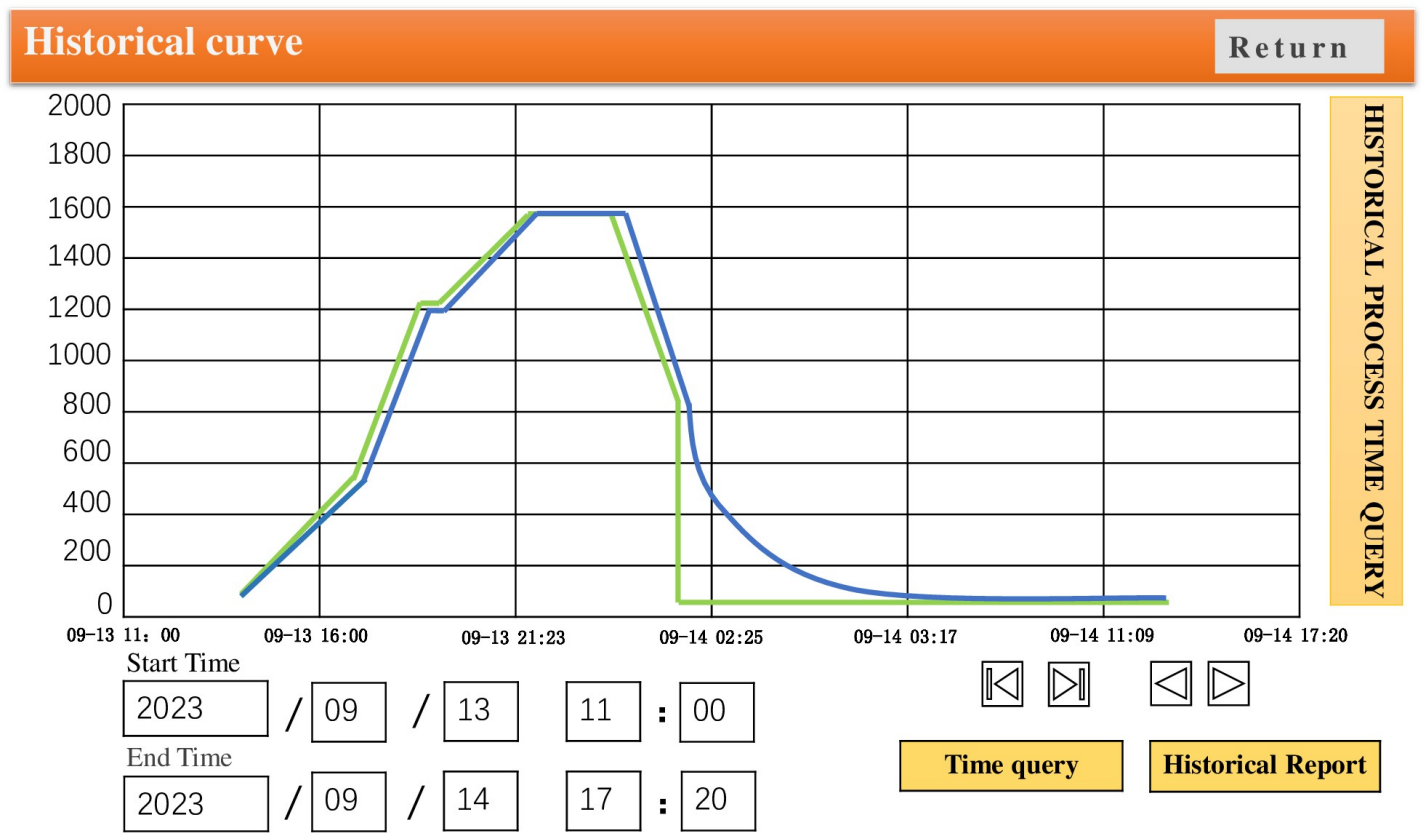
Temperature control curve of vacuum annealing furnace based on fuzzy PID algorithm.

**Fig 20:**
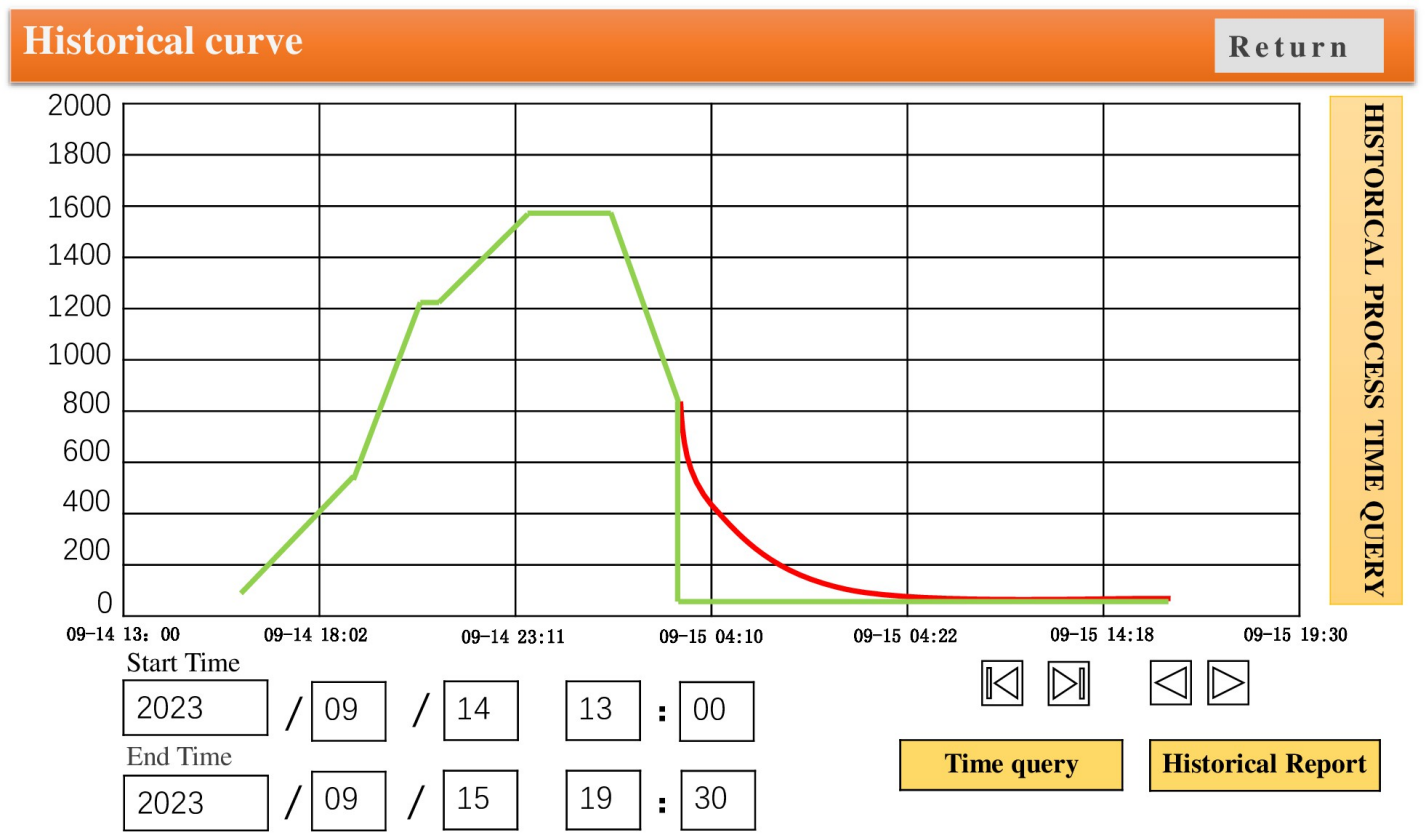
Temperature control curve of vacuum annealing furnace based on GA-Fuzzy PID algorithm.

In order to verify the temperature control effect, GA-Fuzzy PID and Fuzzy PID algorithms were written into the PLC separately. The experimental results are shown in Figs 19 and 20, where the green curve represents the set temperature curve. Through comparative analysis, it is found that there are some deviations between the Fuzzy PID temperature control curve and the temperature setting curve during the heating stage. However, the GA-Fuzzy PID temperature control curve based on GA-Fuzzy PID appears relatively stable during the heating stage, meeting the process requirements of furnace temperature heating and heating system. The experiment shows that the GA-Fuzzy PID algorithm is superior to the Fuzzy PID control algorithm, And the system has good temperature uniformity, meeting the actual annealing requirements of the workpiece.

## 5. Conclusion

This article focuses on the optimization design of the temperature control system of the vacuum annealing furnace. Genetic algorithm is introduced to globally optimize the fuzzy rule table in the fuzzy controller, and programming and simulation are conducted using MATLAB and Simulink environments. The results show that:

(1) The system model of the vacuum annealing furnace temperature control system is built using the GA fuzzy PID control algorithm. The accuracy of the vacuum annealing furnace temperature control system is improved by combining genetic algorithm with fuzzy control.
(2) Through simulation and comparative analysis, it is concluded that the temperature control system of vacuum annealing furnace based on GA fuzzy PID control algorithm has faster response speed, higher control accuracy, stronger stability, and higher temperature control accuracy. Meet the temperature accuracy requirements of vacuum annealing furnace.
(3) By comparing and analyzing the actual temperature curve of the vacuum annealing furnace, based on the fuzzy PID temperature control curve and the set temperature there are some deviations, based on the GA-Fuzzy PID temperature control system, the operation is good and meets the requirements of the metal heat treatment annealing process.

## Supporting information

S1 Data (SLX)
