# Supplementary figures and images for: Design of vacuum annealing furnace temperature control system based on GA-Fuzzy-PID algorithm
Source: PLoS One. 2023 Nov 29;18(11):e0293823. doi: 10.1371/journal.pone.0293823 (PMC10686503; doi:10.1371/journal.pone.0293823)

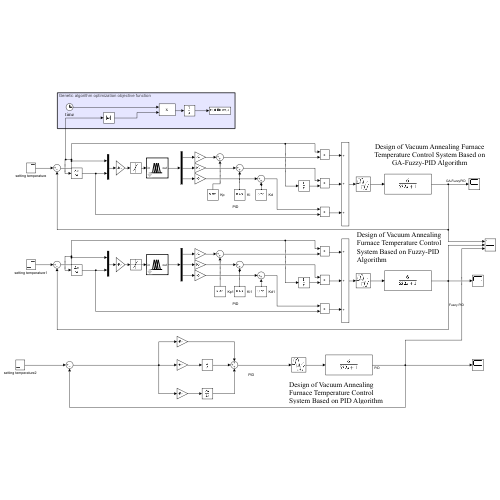

Supplement: S1 Data — (SLX) [file pone.0293823.s001.slx › metadata/thumbnail.png]
